# Amnioreduction for Polyhydramnios in a Consecutive Series at a Single Center: Indications, Risks and Perinatal Outcomes

**DOI:** 10.3390/children11040502

**Published:** 2024-04-22

**Authors:** Arianna Laoreti, Valentina Sala, Daniela Casati, Stefano Faiola, Luigina Spaccini, Irene Cetin, Mariano M. Lanna

**Affiliations:** 1 Fetal Therapy Unit “U. Nicolini”, Buzzi Children’s Hospital, University of Milan, 20154 Milan, Italy; daniela.casati@asst-fbf-sacco.it (D.C.); stefano.faiola@asst-fbf-sacco.it (S.F.); mariano.lanna@asst-fbf-sacco.it (M.M.L.); 2 Department of Woman, Mother and Neonate, Buzzi Children’s Hospital, University of Milan, 20154 Milan, Italyluigina.spaccini@asst-fbf-sacco.it (L.S.); 3 Clinical Genetic Service, Buzzi Children’s Hospital, University of Milan, 20154 Milan, Italy; 4 Fondazione IRCCS Ca’ Granda, Hospital Maggiore Policlinico, University of Milan, 20122 Milan, Italy; irene.cetin@unimi.it

**Keywords:** polyhydramnios, amnioreduction, fetal anomaly, pPROM, preterm labor, fetal death, neonatal death

## Abstract

Pregnancies complicated by severe polyhydramnios are associated with a high rate of underlying fetal anomaly. Amnioreduction may be offered to alleviate maternal symptoms. This is a retrospective study of amnioreductions performed on singleton and twin gestations complicated by symptomatic polyhydramnios between 2010 and 2023 at our tertiary referral center. The indications, procedural techniques and pregnancy and neonatal outcomes were retrieved from an archive database and reviewed with the use of the maternal and child medical record chart, the hospital electronic clinical discharge report and telephone recalls. Our study comprised 86 pregnancies, 65 singletons and 21 twin pregnancies. Fetal anomalies were identified in 79% of cases, mainly gastrointestinal obstructive anomalies; 9.3% of cases were idiopathic. The median gestational age at first amnioreduction was 32.5 weeks, and peri-procedural complications were rare (1 case of placental abruption and 2 cases of preterm delivery). The median gestational age at delivery was 36.5 weeks, with a median prolongation of the pregnancy from the time of first drain until birth of 30 days. Preterm labor < 37 weeks occurred in 48.8% of procedures, with 26.7% of patients delivering before 34 weeks and pPROM < 36 weeks recorded in 23.2% of cases. In conclusion, amnioreduction offered to alleviate maternal symptoms is a reasonably safe procedure with a low complication rate. These pregnancies necessitate management in a tertiary referral center because of their need for a multidisciplinary approach both prenatally and postnatally.

## 1. Introduction

Polyhydramnios is a pregnancy complication characterized by an excessive accumulation of amniotic fluid within the amniotic sac, reported in 1–2% of singleton pregnancies [1,2].

The diagnosis of polyhydramnios is typically made through ultrasound examinations using semiquantitative methods: the single deepest vertical pocket (DVP) of amniotic fluid, with polyhydramnios defined as DVP ≥ 8 cm, or the amniotic fluid index (AFI) with polyhydramnios defined as AFI ≥ 24 cm [2].

Polyhydramnios reflects a final common pathway resulting from various underlying causes, including maternal diabetes, placental tumors and fetal abnormalities. Other causes of polyhydramnios include fetal anemia due to congenital infections and alloimmunization. “Idiopathic” polyhydramnios accounts for approximately 40–50% of cases and should be a diagnosis of exclusion after other potential causes of excessive amniotic fluid have been ruled out. However, in approximately 10% of cases, an underlying fetal abnormality or genetic syndrome is identified after birth, with the risk increasing according to the severity of polyhydramnios [3,4].

There is a well-known association between polyhydramnios and adverse pregnancy and neonatal outcomes, including fetal anomalies, perinatal death and preterm birth [1].

Amnioreduction (AR) has been largely used in the management of twin–twin transfusion syndrome (TTTS) in monochorionic (MC) twin pregnancies before the introduction of placental laser ablation. In this setting, studies have supported the use of amnioreduction as an option to relieve maternal discomfort and to potentially prolong pregnancy, reducing the risk of preterm premature rupture of membranes (pPROM) and preterm labor and improving placental vascular supply [5,6].

Despite the large volume of literature available on AR in twin pregnancies [5,6,7], fewer published studies have analyzed complications of AR in singleton pregnancies, generally reporting a low rate of short-term peri-procedural complications (preterm birth 4%-10%; premature rupture of membranes 0–1%; fetal demise 0–0.4%) [8,9]. With regard to pregnancy outcomes after AR, previous studies reported a median gestational age at birth near term (36–37 weeks), with debated benefits of AR in terms of prolonging the pregnancy and a described increased probability of vaginal delivery and a lower incidence of uterine atony [8,9,10]. Perinatal outcome has been reported to be largely associated with underlying fetal anomaly [8,9,10].

The aim of our study was to review the indications, peri-procedural complications, pregnancy and perinatal outcomes of singleton and twin pregnancies complicated by polyhydramnios, which underwent AR at our institution, contributing to evidence-based practices in counseling and managing pregnancies with polyhydramnios warranting amnioreduction.

## 2. Materials and Methods

This was a descriptive retrospective study of AR procedures performed on singleton and twin pregnancies complicated by polyhydramnios between January 2010 and June 2023 at our Fetal Therapy Unit at Buzzi Children’s Hospital in Milan (Italy).

Cases were identified from an archive database and reviewed with the use of the maternal and child medical record chart, the hospital electronic clinical discharge report and telephone recalls.

All cases underwent a detailed ultrasound assessment of the fetus and placenta performed by a fetal medicine specialist, in addition to maternal screening for diabetes mellitus and common infections.

The degree of polyhydramnios was recorded and categorized as mild, moderate or severe, based on a DVP of 8–11 cm, 12–15 cm or ≥16 cm, respectively [2].

Symptomatic patients who complained of discomfort, abdominal pain or dyspnea were offered AR after detailed counseling and discussions about the recognized risks and benefits related to the procedure.

At our institution, all patients with polyhydramnios and clinical symptoms are offered amnioreduction. Therefore, in our study, it was not possible to obtain an appropriate control group to carry out a comparative analysis.

AR was not performed in women who were in active labor or for whom delivery was considered inevitable in the short term. Twin pregnancies complicated by TTTS were excluded. In twin pregnancies, both monochorionic and dichorionic, we considered amniotic fluid discordance an intertwin difference ≥4 cm in amniotic fluid volumes.

Peri-procedural antibiotic prophylaxis (cephalosporin) and tocolysis was provided. With regard to tocolysis, we utilize 100 mg rectal indomethacin until 24 weeks and intravenous atosiban after 24 weeks (bolus dose 6.75 mg, followed by a continuous high-dose infusion 300 μg/min of Atosiban 37.5 mg/5 mL for three hours, followed by a lower dose of Atosiban 37.5 mg/5 mL subsequent infusion 100 μg/min up to 45 h.

The procedure of trans-abdominal drainage of amniotic fluid was performed using an 18-gauge needle connected to a 60 mL syringe with manual aspiration under continuous ultrasound guidance.

According to our protocol, amniotic fluid samples were sent for fetal karyotyping and arrayCGH in all cases suggestive of a chromosomal/genetic anomaly (e.g., multiple ultrasound anomalies detected at ultrasound or structural/functional defects highly associated with genetic conditions) and for microbiologic studies when an infection was suspected.

Amnioreduction procedure was stopped when a DVP of <8 cm was reached. After 24 h hospitalization, detailed ultrasound scan was performed before discharge, and weekly follow up was scheduled.

Data on maternal pregnancy characteristics and associated fetal/placental anomaly, obstetrical and perinatal outcomes (premature rupture of membranes (pPROM), preterm delivery (PD), placental abruption, chorioamnionitis, fetal and neonatal death) were collected.

Continuous variables are presented as median (interquartile range [IQR]), and categoric data are presented as a number (percentage).

## 3. Results

### 3.1. Maternal and Health Characteristics

During the study period, 86 pregnancies were included, with 21 twin pregnancies, of which 17 were MC diamniotic and 4 dichorionic (DC) with severe polyhydramnios in one fetus. The maternal and pregnancy characteristics are shown in Table 1.

The median maternal age was 34 years (range 30–38.2 years). Most women were nulliparous (71.1%).

The total n ARs performed was 121, with a median number of 1 over their entire gestation (range 1–4). Most women (*n* = 61; 70.9%) underwent one amnioreduction, and the remaining (*n* = 25; 29%) received >1 amnioreduction (17 women received 2 AR; 6 women received 3 AR; 2 women received 4 AR). The procedure was successful in all cases in relieving patients’ symptoms. The median gestational age at first amnioreduction was 32.5 weeks (range 30.0–36 weeks).

### 3.2. Causes of Polyhydramnios

Fetal anomaly was the primary cause of severe polyhydramnios. Sixty-five patients underwent invasive prenatal diagnosis at the timing of amnioreduction or previously during the pregnancy, and thirteen (20.0%) were abnormal. In particular, six pregnancies were complicated by chromosomal anomalies (5 cases of Trisomy 21 and 1 case of Pallister Killian syndrome), and seven cases presented arrayCGH/monogenic anomalies.

Table 2 summarizes the causes of polyhydramnios that required amnioreduction.

We categorized the causes of polyhydramnios of our cohort in macro-groups and defined the specific anomalies within each, as reported in Table 2.

Gastrointestinal obstructive anomalies were the most frequent indication for amniotic fluid drainage. There were 16 cases of isolated duodenal atresia, 10 cases of isolated esophageal atresia and an additional 2 and 3 cases of complicating Trisomy 21. The case of omphalocele described in our cohort was associated with Beckwith–Wiedemann Syndrome and presented visceromegaly in addition to bowel herniation through the abdominal wall defect.

Among the eight pregnancies in the neuromuscular disease group, four of them were complicated by pathogenetic mutations associated with specific syndromes.

1. De novo 9q34.11 microdeletion is associated with a clinical spectrum, including intellectual disability, speech developmental delay, microcephaly, mild dysmorphisms, strabismus and early infantile epileptic encephalopathy.
2. De novo heterozygous pathogenetic variant in ACTA1 gene, associated with nemaline myopathy, a rare genetic muscle disorder characterized by muscle weakness and hypotonia.
3. De novo heterozygous pathogenetic variant in HUWE-1 gene, associated with intellectual disability and epilepsy.
4. De novo heterozygous pathogenetic variant in BICD2 gene, associated with spinal muscular atrophy (SMA).

Among the cases of fetal respiratory diseases, one pregnancy was complicated by a fetal echogenic thoracic lesion detected in the third trimester, which presented rapid growth, increased vascularization and determined a mediastinal shift (differential diagnosis with congenital adenomatoid malformation, pulmonary sequestration, bronchial atresia). Sadly, the neonate died after birth, and the parents denied consent to perform an autopsy. Therefore, anatomopathological diagnosis is lacking.

Fetuses affected by congenital diaphragmatic hernia included in our cohort comprised three cases of right-sided defect with herniation of the liver in the thorax and two cases of left-sided defect with herniation of the stomach and bowel in the chest. A prenatal genetic association was found in two cases (1 Pallister–Killian Syndrome; 1 De novo 17q12 microdeletion), and both neonates died after birth.

In one case included in our cohort, a bronchial atresia was suspected at prenatal ultrasound assessments. Postnatal radiological examinations identified a vascular ring determining an extrinsic airway obstruction and leading to “functional” bronchial atresia.

In six cases, major and minor multiple anomalies were detected (e.g., facial dysmorphism, hepatomegaly, hydrothorax, limb abnormalities, ventriculomegaly, etc.) but no specific syndrome could be defined, despite invasive tests that resulted in normal karyotype and arrayCGH.

In the non-immune-hydrops group, we included two cases of suspected congenital metabolic disease versus RASopathy (confirmatory genetic diagnosis not available at birth). In the remaining three cases of non-immune hydrops, the exome sequencing was negative.

One case included in our cohort was characterized by early-onset severe and refractory unexplained polyhydramnios, which required four amnioreductions, starting from 25 weeks, to relieve maternal symptoms. Bartter syndrome was diagnosed after birth and explained the fetal polyuria and the relevant biochemical abnormality in the amniotic fluid drained, which was characterized by normal sodium and potassium but elevated chloride levels.

Notably, no cases of amnioreduction due to maternal disease, such as diabetes, are included in our cohort. Only in eight cases (9.3%) was there no clear cause for the polyhydramnios in the perinatal period, and they were classified as idiopathic.

### 3.3. Procedural Characteristics and Complications

The procedural characteristics and complications are reported in Table 3.

The median pre-procedural maximal deepest vertical pocket (DVP) of amniotic fluid was 13 cm (range 12–14 cm). In 84.7% of cases, the polyhydramnios requiring amnioreduction was classified as moderate or severe, while only in 15.3% of cases was mild. The median amount of fluid removed at each procedure was 2000 mL (range 1700–2662 mL), and the median measure of the deepest vertical pocket of amniotic fluid after the procedure was 7 cm (range 6.7–8 cm).

The peri-procedural complications occurring <48 h post-procedure were recorded. No cases of chorioamnionitis or sepsis were recorded among the study cohort. Only one case (0.8%) of placental abruption occurred at 34.1 weeks after a second amnioreduction performed for polyhydramnios caused by a suspected fetal neurological issue (unknown etiology, likely genetic anomaly in consanguineous parents). The neonate died after birth.

Preterm delivery occurred within 48 h after the procedure in 2 cases/121 (1.65%) cases of amnioreductions performed at 36 weeks and 33.6 weeks, respectively. These cases necessitating delivery at <48 h included one case of pPROM at 36 weeks after amnioreduction for fetal thoracic mass at rapid growth with mediastinal shift. The neonate died after birth. The second case was an amnioreduction performed at 33.6 weeks due to idiopathic polyhydramnios in which emergency cesarean delivery was indicated after 24 h due to non-reassuring fetal heart rate tracing.

### 3.4. Pregnancy and Neonatal Outcomes

Pregnancy and neonatal outcomes are presented in Table 4.

Median gestational age at delivery was 36.5 weeks (IQR 34.0–38.0). The rate of cesarean deliveries was high in our cohort (69.9%), with one-third of cesarean deliveries being conducted before 34 weeks.

The median interval between the first amnioreduction and delivery was 30 days (IQR 17–41, range 1–120). Preterm premature rupture of membranes < 36 weeks was recorded in 20/86 cases (23.2%), and the median interval between the first procedure and pPROM was 16 days (IQR 7.5–26, range 0–52).

Fetal demise occurred in three cases (3.5%).

1. A DC twin at 22 weeks and 3 days GA where polyhydramnios was related to fetal non-immune hydrops (suspected congenital metabolic disease versus RASopathy), and fetal demise occurred two weeks after procedure.
2. A trisomy 21 fetus at 38 weeks, after 2 AR performed for esophageal obstruction.
3. A polymalformated fetus with hydrothorax, Binder-like phenotype and hepatomegaly (unknown etiology, suspected metabolic disease) with demise occurring at 30 weeks GA, 6 days after AR.

Neonatal death occurred in 15 cases (18%) out of 83 liveborn who underwent AR; in 7 cases, a known or suspected chromosomal/genetic etiology was recorded; 2 cases were hydropic neonates; in 6 cases, death occurred because of severe respiratory compromise or severe prematurity.

## 4. Discussion

Our study presented indications and pregnancy outcomes in 86 pregnancies (65 singletons and 21 twin pregnancies) who underwent 121 amnioreductions due to symptomatic polyhydramnios in our tertiary referral Centre of Fetal Diagnosis and Therapy at Buzzi Children’s Hospital.

The first finding that warrants consideration in our cohort is the high prevalence of structural or functional fetal/placental abnormality (79%) and the high incidence of fetuses who died perinatally (1 in 5). Previous studies have demonstrated an association between the severity of the polyhydramnios and the likelihood of a fetal abnormality [11].

The Society of Maternal and Fetal Medicine (SMFM), in its Consult on polyhydramnios, stated that polyhydramnios identification should prompt an exploration of its underlying etiology and, importantly, “idiopathic” polyhydramnios should be a diagnosis of exclusion [2].

Indeed, although the cause of polyhydramnios may be unexplained prenatally, an etiology may become manifest after birth. In cases of moderate and severe polyhydramnios, the rate of fetal abnormality detected prenatally is up to 15% and 40%, respectively, with an additional chance of postnatal diagnosis of 2% and 10%, respectively [2]. In our cohort, 84.7% of cases of polyhydramnios requiring amnioreduction were classified as moderate or severe, while only 15.3% of cases were mild. This “high-risk” population may reflect the high proportion of anomalies identified and the relatively small amount of “idiopathic” polyhydramnios. All patients in our series received detailed ultrasound scans by fetal medicine practitioners, and relevant cases were managed in close collaboration with clinical geneticists who performed neonatal examinations when indicated, leading to an increase in the postnatal detection rate of anomalies. Our finding is in line with a previously published series in which, amongst 138 pregnancies with moderate–severe polyhydramnios, a fetal anomaly was identified in 77.5% of cases and in 84.9% of total amnioreductions performed [9].

Lastly, no cases of maternal disease, such as pre-pregnancy or gestational diabetes, were comprised in our cohort. Since amnioreduction was indicated only in cases of severe maternal discomfort, dyspnea or both, it may be possible that mild cases of diabetes-related polyhydramnios are not included in our cohort; this was also in line with strict follow-up of these pregnancies in high-risk settings and universal screening of gestational diabetes in our cohort, possibly limiting the incidence and severity of polyhydramnios in this specific population.

Our study confirms the finding that amnioreduction for polyhydramnios in singleton and twin pregnancies is a safe procedure. The complication rate was low, especially when considering the intrinsic risks associated with severe polyhydramnios. As previously illustrated, polyhydramnios may result in maternal discomfort, pPROM, preterm labor, fetal malposition, placental abruption, non-reassuring fetal heart rate tracings and postpartum hemorrhage due to uterine atony related to overdistension [1,2,12]. These complications also heighten the probability of cesarean delivery and admission of the neonate to the intensive care unit, also in cases with mild idiopathic polyhydramnios [2].

If polyhydramnios interferes with the patient’s daily activities, in the last three decades, amnioreduction has been recommended in order to alleviate maternal discomfort and possibly reduce the rate of indicated preterm births for maternal complications [4,13].

There is no consensus on the optimal approach for managing polyhydramnios, including the amount of fluid to remove, the frequency of the procedure, the speed of fluid removal or the use of tocolytic and antibiotics. Notably, limited published series exist on amnioreduction, and some of them include data from twin pregnancies complicated by twin-to-twin transfusion syndrome (TTTS), where the pathophysiological condition differs significantly [5].

In our study, peri-procedural complications within 48 h occurred rarely, with no cases of chorioamnionitis or sepsis recorded and only one case (0.8%) of placental abruption after a second amnioreduction. Delivery occurred within 48 h after the procedure, which was only in 1.65% of cases. These results are in line with previous reports [8,9,10]. Erfani et al., in 2019, reported peri-procedural complications in 66 amnioreductions in singleton pregnancies, and no cases of infections or pPROM were recorded, while 10.6% of cases experienced preterm delivery within 48 h of amnioreduction [10]. Similar data were reported in the largest case series of amnioreduction by Dickinson et al., where amongst 271 procedures, only 1.1% experienced a pPROM, and 4.1% of cases were delivered within 48 h [9].

With regard to pregnancy outcome, in our study, the median gestational age at delivery was 36.5 weeks, with a median interval of 16 days from first procedure to pPROM and a median prolongation of the pregnancy from the time of first AR until birth of 30 days. Similar data were found in the series published by Dickinson et al., where the median interval from the first procedure until delivery was 26 days, with a median gestational age at delivery of 36.4 weeks [9].

Nevertheless, caution should be used when interpreting the possible positive role of amnioreduction in prolonging gestation, similar to that observed in TTTS pregnancies treated with AR [5,6], since the exact impact of the procedure on these outcomes remains uncertain. Recently, Soni et al. conducted a retrospective review of singleton pregnancies with moderate-severe polyhydramnios due to fetal anomalies, comparing those that underwent amnioreduction versus those that were expectantly managed [14]. The authors found that amnioreduction had a low complication rate (preterm delivery <48 h in 5.4% cases) but did not provide benefit in terms of protracting the pregnancy. However, patients undergoing AR had an earlier onset of polyhydramnios and a higher amount of amniotic fluid compared to those who did not desire AR. Moreover, the patients in the amnioreduction group had significantly higher rates of vaginal delivery (49.4% versus 30.5%) and statistically significant lower rates of uterine atony (2.4% versus 13.7%) [14]. Given the high rate of cesarean deliveries broadly reported in the literature in pregnancies complicated by polyhydramnios (69.9% in our cohort), this finding merits special consideration and argues in favor of the execution of AR in symptomatic patients.

The present study also confirms the relevant rate of stillbirth (3.5%) and neonatal death (18%) in pregnancies complicated with polyhydramnios requiring amnioreduction. This finding is consistent with the already highlighted high incidence of fetal anomaly as underlying etiology and supports the recommendation of referring cases with severe polyhydramnios at tertiary centers for prenatal management and delivery due to the significant risk that fetal anomalies may be present at birth, even if not prenatally identified.

In addition, despite the perinatal outcome being mainly determined by the primary fetal anomaly, it is conceivable that cases requiring early neonatal surgery (principally gastrointestinal and respiratory diseases) may benefit from protraction of pregnancy possible due to amnioreduction, especially in terms of anesthesiologic risks and peri-operative care. Multidisciplinary-team management of these cases, involving obstetricians, geneticists, neonatologists and pediatric surgeons, ensures the best chances of care and support in these pregnancies.

The main strengths of this study are represented by the size of the study cohort, the uniform amnioreduction protocol performed at a single center by fetal medicine practitioners and the availability of neonatal follow-up, which permitted postnatal diagnosis, thus increasing our detection rate of associated anomalies.

Due to the unicity of this study population in which polyhydramnios was largely moderate–severe and the consequence of fetal anomalies, two limitations could be identified. First, in our study, it was not possible to obtain an appropriate control group to carry out a comparative analysis since all symptomatic patients with polyhydramnios are offered amnioreduction in our Fetal Therapy Unit. This could also be explained by the fact that less severe cases, therefore not associated with maternal symptoms, are not routinely referred to our Unit. Secondly, the high prevalence of fetal anomalies may represent a bias since the findings of this study may not be fitting to a general population of pregnancies in which polyhydramnios are not related to fetal diseases. Nevertheless, it may be assumed, based on previously published studies, that most of these results will be appropriate in all cases requiring amnioreduction, regardless of the primary cause determining polyhydramnios. The most suitable comparison group would be a control population of pregnancies with idiopathic moderate–severe polyhydramnios, though difficult to obtain because the vast majority of idiopathic polyhydramnios patients have mild polyhydramnios and will not likely require an AR.

In addition, we acknowledge the limitations of the retrospective study design, including the absence of robust and systematical data on cervical length pre- and post-procedure and the additional treatment for threatened preterm labor.

## 5. Conclusions

Pregnancies complicated by moderate–severe polyhydramnios requiring amnioreduction are associated with a high rate of underlying fetal or placental anomaly. Amnioreduction offered to alleviate maternal symptoms has a useful role and represents a procedure with a reasonably low rate of potential complications. These pregnancies warrant delivery in a tertiary referral center not only for the risk of preterm delivery but also for the need for a multidisciplinary approach both prenatally and postnatally.

Future research should consider differences in the amnioreduction approaches (amount of fluid removed, frequency of the procedure, the speed of fluid removal, use of tocolytic and antibiotics), the potential prognostic role of cervical length in polyhydramnios, its impact on latency from drainage until birth and better define the ability of amnioreduction to protract the pregnancy, also in relation to cervical length.

## Figures and Tables

**Table 1:** Maternal and pregnancy characteristics.

Variable	Measure
Maternal age, years	34 (30–38.2)
Parity	0 (0–1)
ART *, *n* (%)	12/86 (13.9%)
Twin pregnancy, *n* (%)	21/86 (24.4%)
Gestational age at first AR, weeks	32.5 (30.0–36.0)
Number of AR ** per woman, *n*	1 (1–4)

Data are given as median (interquartile range); * Assisted Reproductive Technology. ** Amnioreduction.

**Table 2:** Causes of polyhydramnios that required amnioreduction.

Cause of Polyhydramnios	*n* (%)
Fetal/placental abnormality	68/86 (79%)
Gastrointestinal -Duodenal obstruction -Esophageal atresia -Gastric transposition -Pyloric obstructive membrane -Omphalocele	34 (39.5%) 18 (2 Trisomy 21) 13 (3 Trisomy 21) 1 1 1 (Beckwith–Wiedemann Syndrome)
Neuromuscular disease	8 (9.3%) (4 genetic syndromes)
Fetal respiratory disease -Congenital diaphragmatic hernia -Bronchial atresia (vascular ring) -Thoracic mass with mediastinal shift	7 (8.1%) 5 (1 Pallister–Killian Syndrome; 1 De novo 17q12 microdeletion) 1 1
Placental chorioangioma	6 (6.9%)
Non-Immune Hydrops	5 (5.8%)
Multiple anomalies	6 (6.9%)
Osmotic diuresis	1 (1.2%) (Bartter Syndrome)
Twin pregnancies	11 (12.8%) (Twin discordance of amniotic fluid)
Idiopathic	8 (9.3%)

**Table 3:** Procedural characteristics and complications.

Variable	Measure
Procedural characteristics Pre-procedural maximal DVP of amniotic fluid, cm	13 (12–14)
Post-procedural maximal DVP of amniotic fluid, cm	7 (6.7–8)
Volume removed per AR, mL	2000 (1700–2662)
Post-procedural complications (<48 h)	
Infections, *n* (%)	0
Placental abruption, *n* (%)	1/121 (0.8%)
pPROM, *n* (%)	1/121 (0.8%)
Delivery, *n* (%)	2/121 (1.65%)

Data are given as median (interquartile range).

**Table 4:** Pregnancy-neonatal outcomes.

Variable	Measure
Gestational age at delivery, weeks	36.5 (34.0–38.0)
Birthweight, g	2470 (1877–2732)
Preterm labor < 34 weeks, *n* (%)	23/86 (26.7%)
Preterm labor < 37 weeks, *n* (%)	42/86 (48.8%)
Time interval first procedure–labor, days	30 (IQR 17–41; range 1–120)
pPROM < 36 weeks, *n* (%)	20/86 (23.2%)
Time interval first procedure–pPROM, days	16 (IQR 7.5–26; range 0–52)
Intrauterine fetal death, *n* (%)	3/86 (3.5%)
Neonatal death, *n* (%)	15/83 * (18%)

* Number of liveborn after exclusion of IUDF (Intra Uterine Fetal Death).

## Data Availability

The data presented in this study are available on request from the corresponding author. The data are not publicly available due to restrictions, e.g., privacy or ethical.
